# Going deeper in the automated identification of Herbarium specimens

**DOI:** 10.1186/s12862-017-1014-z

**Published:** 2017-08-11

**Authors:** Jose Carranza-Rojas, Herve Goeau, Pierre Bonnet, Erick Mata-Montero, Alexis Joly

**Affiliations:** 1 Costa Rica Institute of Technology, Cartago, Costa Rica; 2 CIRAD-Amap, Montpellier, France; 3 INRIA, Montpellier, France

**Keywords:** Biodiversity informatics, Computer vision, Deep learning, Plant identification, Herbaria

## Abstract

**Background:**

Hundreds of herbarium collections have accumulated a valuable heritage and knowledge of plants over several centuries. Recent initiatives started ambitious preservation plans to digitize this information and make it available to botanists and the general public through web portals. However, thousands of sheets are still unidentified at the species level while numerous sheets should be reviewed and updated following more recent taxonomic knowledge. These annotations and revisions require an unrealistic amount of work for botanists to carry out in a reasonable time. Computer vision and machine learning approaches applied to herbarium sheets are promising but are still not well studied compared to automated species identification from leaf scans or pictures of plants in the field.

**Results:**

In this work, we propose to study and evaluate the accuracy with which herbarium images can be potentially exploited for species identification with deep learning technology. In addition, we propose to study if the combination of herbarium sheets with photos of plants in the field is relevant in terms of accuracy, and finally, we explore if herbarium images from one region that has one specific flora can be used to do transfer learning to another region with other species; for example, on a region under-represented in terms of collected data.

**Conclusions:**

This is, to our knowledge, the first study that uses deep learning to analyze a big dataset with thousands of species from herbaria. Results show the potential of Deep Learning on herbarium species identification, particularly by training and testing across different datasets from different herbaria. This could potentially lead to the creation of a semi, or even fully automated system to help taxonomists and experts with their annotation, classification, and revision works.

## Background

For several centuries, botanists have collected, catalogued and systematically stored plant specimens in herbaria. These biological specimens in research collections provide the most important baseline information for systematic research [1]. These physical specimens ensure reproducibility and unambiguous referencing of research results relating to organisms. They are used to study the variability of species, their phylogenetic relationship, their evolution, and phenological trends, among others. The estimated number of specimens in Natural History collection is in the 2–3 billion range [2]. There are approximately 3000 herbaria in the world, which have accumulated around 350,000,000 specimens [3], i.e., whole plants or plant parts usually in dried form and mounted on a large sheet of paper.

Large scale digitization of specimens is therefore crucial to provide access to the data that they contain [4]. Recent national and international initiatives such as iDigBio [5] or e-ReColNat started ambitious preservation plans to digitize and facilitate access to herbarium data through web portals accessible to botanists as well as the general public. New capacities such as specimen annotation [6] and transcription [7] are offered in these portals. However, it is estimated that more than 35,000 species not yet described and new to science have already been collected and are stored in herbaria [8]. These specimens, representing new species, remain undetected and undescribed because they may be inaccessible, their information is incomplete, or the necessary expertise for their analysis is lacking. These new species are then unnoticed, misplaced, or treated as unidentified material. Thousands and thousands of sheets are still not identified at the species level while numerous sheets should be reviewed and updated following more recent taxonomic knowledge. These annotations and revisions require such a large amount of work from botanists that it would be unfeasible to carry them out in a reasonable time.

Computer vision approaches based on the automated analyses of these sheets may be useful for such species identification tasks. Furthermore, such automated analysis could also help botanists in the processes of discovering and describing new species among the huge volume of stored herbarium specimens. As a result, evolutionary and ecological studies could be strongly accelerated due to the quick access to the most interesting specimens of a particular group of species. A tool that, based on herbarium sheet images across multiple collections world wide, finds the plant specimens more similar to a candidate would be of great help for taxonomists and botanists working at herbaria. However, this is still a very challenging objective. Because specimens are mounted on sheets assuming that they will be used and visually inspected by humans, the amount of visual noise present in this type of image is very high for fully automated computer vision processing. Nevertheless, in the last five years, deep learning has become a promising tool to handle extremely complex computer vision tasks. Additionally, online portals of ambitious initiatives such as iDigBio already provide access to more than 14 million herbarium images [9] that are particularly useful for deep learning approaches [10]. Thus, it is now possible to use images of herbaria thanks to current advances in machine learning and initiatives such as iDigBio.

With this study we aim to answer three questions: (i) are herbarium images useful for herbaria-only classification using deep learning? (ii) Can a deep learning model learn relevant features from herbarium images and be successfully used for transfer learning to deal with field images? (iii) And finally, can herbarium images from one region of the world, be used for transfer learning on a herbarium dataset from another region, especially for a region under-represented in terms of collected data?

The following are the main contributions of this research:

- New datasets of herbaria properly curated for machine learning purposes, including one small dataset (255 species, 7.5 ∼k images) and one large dataset (1204 species, 260 ∼k images).
- Demonstration of the feasibility of implementing an identification system for herbarium data at a realistic scale, i.e., with 30 times more species than previous studies in the literature [11].
- Experiments to study the usage of herbaria for transfer learning to field photos.
- Demonstration of the potential of using herbaria from one region of the world for transfer learning to another region, with different species.

To our knowledge, this is the first study on theautomated analysis of herbarium collections with a large number of sheets and the first one using deep learning techniques. The rest of this manuscript is organized as follows: “Related work” section presents relevant related work. “Methods” and “Experiments and results” sections cover experiment design and the results obtained, respectively. “Discussion and conclusions” section presents conclusions and summarizes future work.

## Related work

Among the diverse methods used for species identification, Gaston et al. [12] discussed in 2004 the potential of automated approaches typically based on machine learning and multimedia analysis methods. They suggested that, if the scientific community is able to (i) overcome the production of large training datasets, (ii) more precisely identify and evaluate the error rates, (iii) scale up automated approaches, and (iv) detect novel species, it will then be possible to initiate the development of a generic automated species identification system that could open opportunities for work in biological and related fields. Since the question raised by Gaston et al. (“Automated species identification: why not?”), considerable work has been done on the development of automated approaches for plant species identification, mostly based on computer vision techniques (e.g. [13–19]). A recent and exhaustive review of plant identification using computer vision techniques has been published by Wäldchen et al. [20]. Some of these results were integrated in effective web or mobile tools and have initiated close interactions between computer scientists and end-users such as ecologists, botanists, educators, land managers and the general public. One remarkable system in this domain is the LeafSnap application [21], focused on a few hundred tree species of North America and on the contour of leaves alone. This was followed a few years later by other applications such as Folia [22] and the popular Pl@ntNet application [23] that now accounts for millions of users all around the world.

However, very few studies have attempted to use herbaria for automated plant classification. So far, most of the biodiversity informatics research related to herbaria has focused on digitization of their collections [24]. Thiers et al. [25] use a small dataset of the genus Stemonoporus, endemic to Sri Lanka, that contains a total of 17 species and 79 images. They extracted morphometric features such as leaf length, width, area and perimeter. The reported accuracy for species identification is 85%. Unger et al. [11] use Support Vector Machine (SVM) with Fourier features and morphometric measures to identify species in two test sets, one with 26 species, the other with 17, in each case using 10 images per species, with respective accuracy of 73.21 and 84%. In all these previous studies, the amount of data used was relatively small and restricted to few tens of species. To have more conclusive results and to plan more realistic scenarios, our work focuses on large datasets. Actually, for a given flora from one region, thousands of species can potentially be expected. Therefore, numerous confusions can be encountered not only among species related to a same genus, for instance, but also across genera that share some similar visual patterns.

Besides species identification, some other studies have attempted to automatically extract characters or attributes from herbarium data. It was demonstrated in [26] that leaf characters can be automatically extracted using a hand-crafted workflow of state-of-the-art image analysis techniques. It is likely that such ad-hoc workflow would not generalize well to other herbarium data. Moreover, it is not applicable to the other parts of the plant such as flowers, fruits, etc. More recently, Dominik Tomaszewski et al. [27] aimed at determining whether leaf shape changes during the drying process (using elliptic Fourier analysis combined with principal component analysis as well as manual measurements). The results indicate that the preservation process of pressing and drying plants for herbarium purposes causes changes in leaf shape so that they suggest that shape analyses should be performed on datasets containing only one of the leaf types (dried and fresh leaves).

On the deep learning side, Yosinski et al. [28] study the effects of progressive transfer learning. They conclude that the first layers of the model relate to generic features and help a lot during the transfer itself. However, this is not focused on a particular domain, leaving open the question of how much transfer learning changes if the dataset used for it is from a specific domain or of a similar domain. In particular for plant recognition, it remains to be seen if a very specific domain dataset, such as herbaria, can be used to learn and fine tune with other similar, related datasets, such as field images of plants.

## Methods

The following subsections describe the deep learning model used in the experiments, the transfer learning approach, the datasets, and the provisions made to avoid biases and to pre-process all datasets.

### Deep learning model

We focused our experiments on the use of Convolution Neural Networks [29], which have been shown to considerably improve the accuracy of automated plant species identification compared to previous methods [20, 30, 31]. More generally, Convolution Neural Networks (CNNs) recently received much attention because of the impressive performance they achieved in the ImageNet classification task [32]. The main strength of these technologies comes from their ability to learn discriminant visual features directly from the raw pixels of the images without falling into the trap of the curse of dimensionality, referring to the exponentially increase of the model variables as the dimensionality grows [10]. This is achieved by stacking multiple *convolutional layers*, i.e., the core building blocks of a CNN. A convolutional layer basically takes images as input and produces as output *feature maps* corresponding to different convolution kernels, while looking for different visual patterns.

Looking at the impressive results achieved by CNN’s in the 2015 and 2016 edition of the international PlantCLEF challenge [31, 33] on species identification, there is no doubt that they are able to capture discriminant visual patterns of the plants in a much more effective way than previously engineered visual features. In particular, we used an extended version of the GoogleNet model [34] that is a very deep CNN that stacks several so-called inception layers. We extended the base version with Batch Normalization [35] which has been proven to speed up convergence and limits overfitting and with a Parametric Rectified Linear Unit (PReLU) activation function [36] instead of the traditional Rectified Linear Unit (ReLU).

**Fig. 1:**
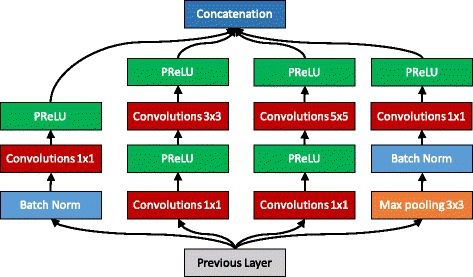
Modified Inception module using PReLU and Batch Normalization

Table 1 shows the modified GoogleNet model with the batch normalization added outside the Inception modules. Just like the original GoogleNet, the model is comprised of several inception modules, however Batch Normalization is added inside each inception module for faster convergence right after each pooling layer. Figure 1 shows how the modified Inception module is comprised. The model was implemented by using the Caffe framework [37]. A batch size of 16 images was used for each iteration, with a learning rate of 0.0075 with images of 224×224 resolution. Simple crop and resize data augmentation was used with the default settings of Caffe.

**Table 1:** GoogleNet architecture modified with Batch Normalization

Type	Patch size / Stride	Output size	Depth	Params	Ops
Convolution	7 ×7/2	112 ×112×64	1	2.7K	34M
Max pool	3 ×3/2	56 ×56×64	0		
Batch norm		56 ×56×64	0		
LRN		56 ×56×64	0		
Convolution	3 ×3/1	56 ×56×192	2	112K	360M
Max pool	3 ×3/2	28 ×28×192	0		
Batch norm		28 ×28×192	0		
LRN		28 ×28×192	0		
Inception (3a)		28 ×28×256	2	159K	128M
Inception (3b)		28 ×28×480	2	380K	304M
Max pool	3 ×3/2	14 ×14×480	0		
Batch norm		14 ×14×480	0		
Inception (4a)		14 ×14×512	2	364K	73M
Inception (4b)		14 ×14×512	2	437K	88M
inception (4c)		14 ×14×512	2	463K	100M
Inception (4d)		14 ×14×528	2	580K	119M
Inception (4e)		14 ×14×832	2	840K	170M
Max pool	3 ×3/2	7 ×7×832	0		
Batch norm		7 ×7×832	0		
Inception (5a)		7 ×7×832	2	1072K	54M
Inception (5b)		7 ×7×1024	2	1388K	71M
Avg pool	7 ×7/1	1 ×1×1024	0		
Batch norm		1 ×1×1024	0		
Linear		1 ×1×10000	1	1000K	1M
Softmax		1 ×1×10000	0		

### Transfer learning

Transfer learning is a powerful paradigm used to overcome the the lack of sufficient domain-specific training data. Deep learning models actually have to be trained on thousands of pictures per class to converge to accurate classification models. It has been shown that the first layers of deep neural networks deal with generic features [28] so that they are generally usable for other computer vision tasks. Consequently they can be trained on arbitrary training image data. Moreover, the last layers themselves contain more or less generic information transferable from one classification task to another one. These layers are expected to be more informative for the optimization algorithm than a random initialization of the weights of the network. Therefore, a common practice is to initialize the network by pre-training it on a big available dataset and then fine-tune it on the scarcer domain-specific data. Concretely, the methodology we used in our experiment for transferring knowledge from dataset *A* to dataset *B* is the following:

1. The network is first trained from scratch on dataset *A* by using a multinomial logistic regression on top of the SOFTMAX layer and the linear classification layer.
2. The linear classification layer used for dataset *A* is then replaced by a new one aimed at classifying the classes in *B*. It is initialized with random weights.
3. The other layers are kept unchanged so as to initialize the learning of dataset *B* with the weights learned from *A*.
4. The network is trained on the images in *B*.

### Herbarium data

**Fig. 2:**
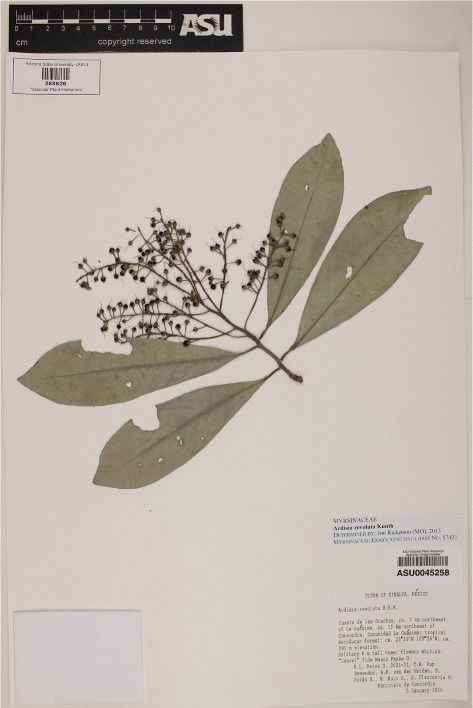
*Ardisia revoluta* Kunth herbarium sheet sample taken from Arizona State University Herbarium

Herbarium data used in the experiments comes from the iDigBio portal, which aggregates and gives access to millions of images for research purposes. As illustrated in Fig. 2, typical herbarium sheets result in a significantly affected visual representation of the plant, with a typical monotonous aspect of brown and dark green content and a modified shape of the leaves, fruits or flowers due to the drying process and aging. Moreover, the sheets are surrounded by handwritten/typewritten labels, institutional stamps, bar codes and even reference colour bar patterns for the most recent ones. Whereas all of these items are very useful for botanists, they generate a significant level of noise from a machine learning point of view. This research aims at assessing if these images can be handled by deep learning algorithms as suggested in [38]. We focus on species classification.

### Datasets

**Fig. 3:**
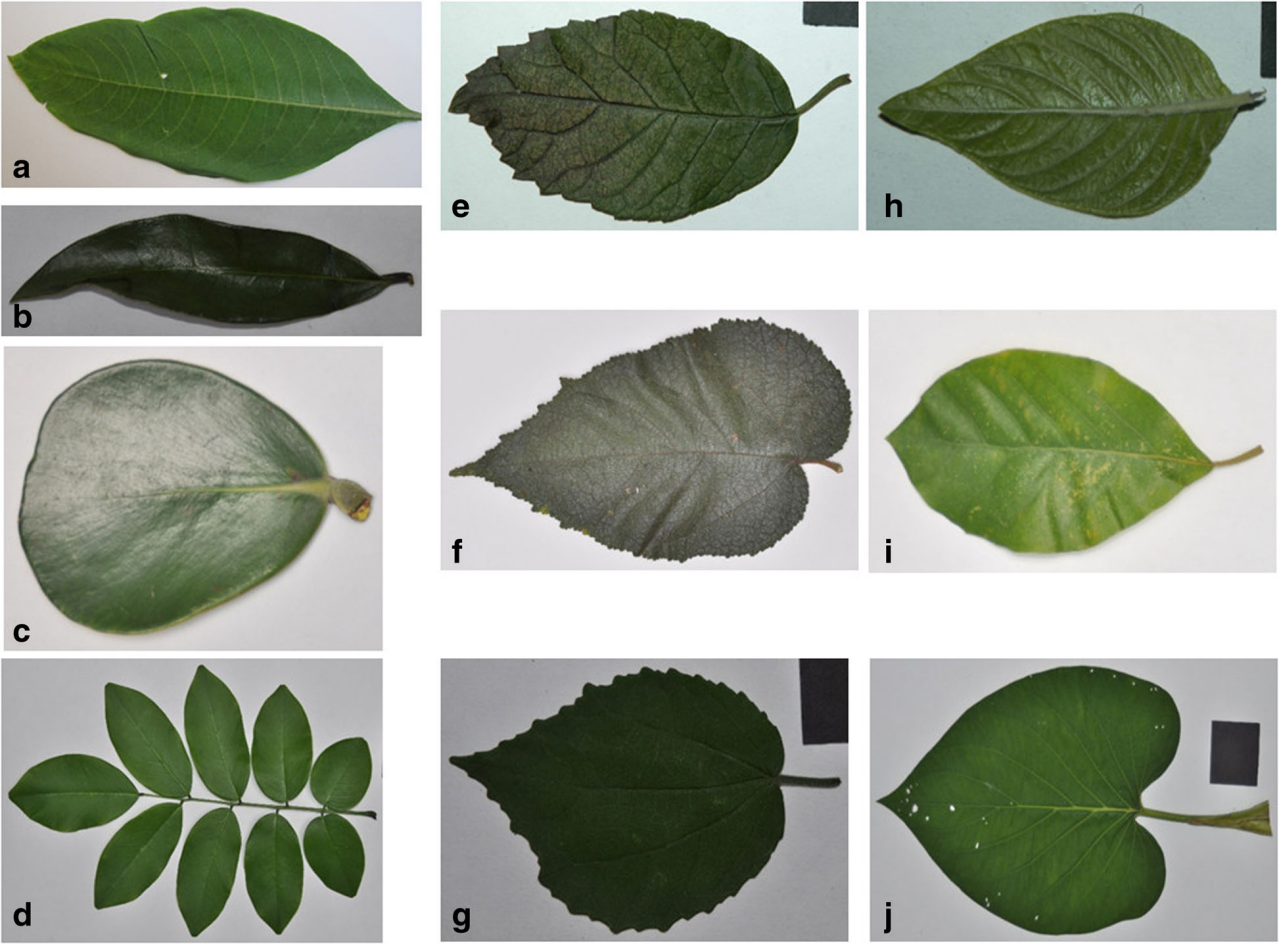
Ten leaf-scan images of different species used in the CRLeaves (CR) dataset: **a** *Acnistus arborescens* (L.) Schltdl, **b** *Brunfelsia nitida* Benth, **c** *Clusia rosea* Jacq, **d** *Dalbergia retusa* Hemsl, **e** *Ehretia latifolia* Loisel. ex A.DC, **f** *Guazuma ulmifolia* Lam, **g** *Malvaviscus arboreus* Cav, **h** *Pentas lanceolata* (Forssk.) Deflers, **i** *Persea americana* Mill, **j** *Piper auritum* Kunth

We used five datasets in this research. Two of them use herbarium sheet images from iDigBio; two more use non-dried plant pictures from Costa Rica and France; additionally, ImageNet weights were used to pre-train the deep learning model. We only used the weights of a pre-trained model on ImageNet, not the dataset itself. ImageNet is a well known generalist dataset which is not dedicated to plants, for this reason we didn’t not use directly the data of this dataset. Table 2 shows the different datasets. The following paragraph explains each dataset and the associated acronyms used throughout this paper:

- CRLeaves (CR): the Costa Rica Leaf Scan Dataset (CRLeaves) includes a total of 255 species from the Central Plateau in Costa Rica. It consists of 7262 images digitized by the National Museum of Costa Rica and the Costa Rica Institute of Technology [39]. Figure 3 shows a random sample of this dataset. This is an unbalanced dataset.

**Table 2:** Datasets used in this research

Name	Acronym	Source	Type	# of Images	# of Species/Classes
CRLeaves	CR	Costa Rica Central Plateau	Leaf Scans	7 262	255
Herbarium255	H255	iDigBio	Herbarium Sheets	11,071	255
PlantCLEF2015	PC	French Mediterranean	In-The-Wild / All organs	113,205	1000
Herbarium1K	H1K	iDigBio	Herbarium Sheets	253,733	1 204
ImageNet	I	ImageNet Challenge	Generic Images	1M	1000
- Herbarium255 (H255): this dataset includes 255 species that match 213 of the species present in the CRLeaves dataset. It uses the iDigBio [5] database and has a total of 11,071 images. Figure 4 shows a random sample of pictures from this dataset. This is an unbalanced dataset.

**Fig. 4:**
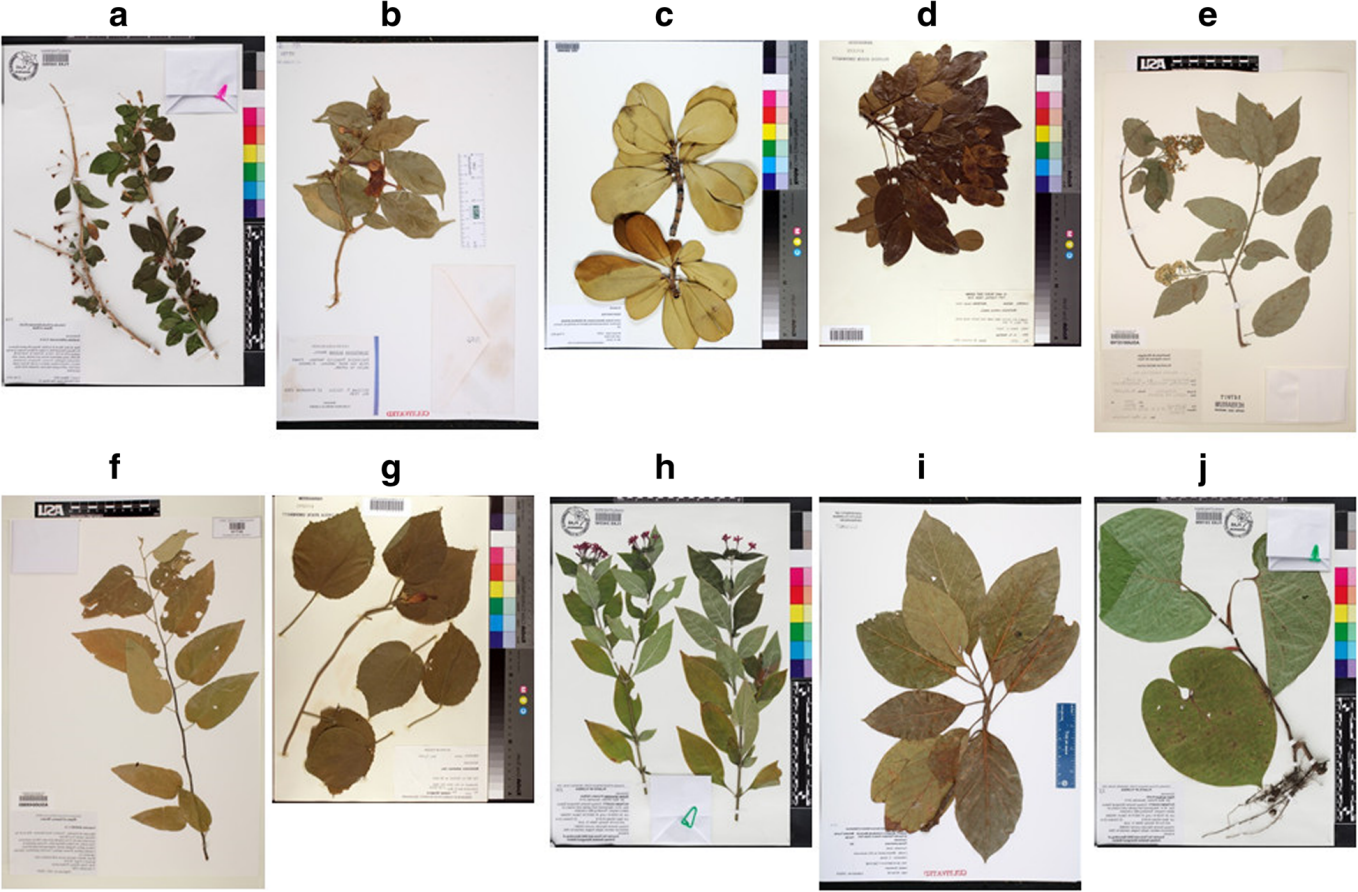
Ten herbarium sheet images of different species used in the H255 dataset: **a** *Acnistus arborescens* (L.) Schltdl, **b** *Brunfelsia nitida* Benth, **c** *Clusia rosea* Jacq, **d** *Dalbergia retusa* Hemsl, **e** *Ehretia latifolia* Loisel. ex A.DC, **f** *Guazuma ulmifolia* Lam, **g** *Malvaviscus arboreus* Cav, **h** *Pentas lanceolata* (Forssk.) Deflers, **i** *Persea americana* Mill, **j** *Piper auritum* Kunth
- PlantCLEF (PC): this is the dataset used in the 2015 PlantCLEF competition. It includes 1000 species, 91,759 images for training, and 21,446 images for testing [31]. Images are from the field and have many organs present. Most images are from the French Mediterranean region. Figure 5 shows a random sample of this dataset. This is also an unbalanced dataset.

**Fig. 5:**
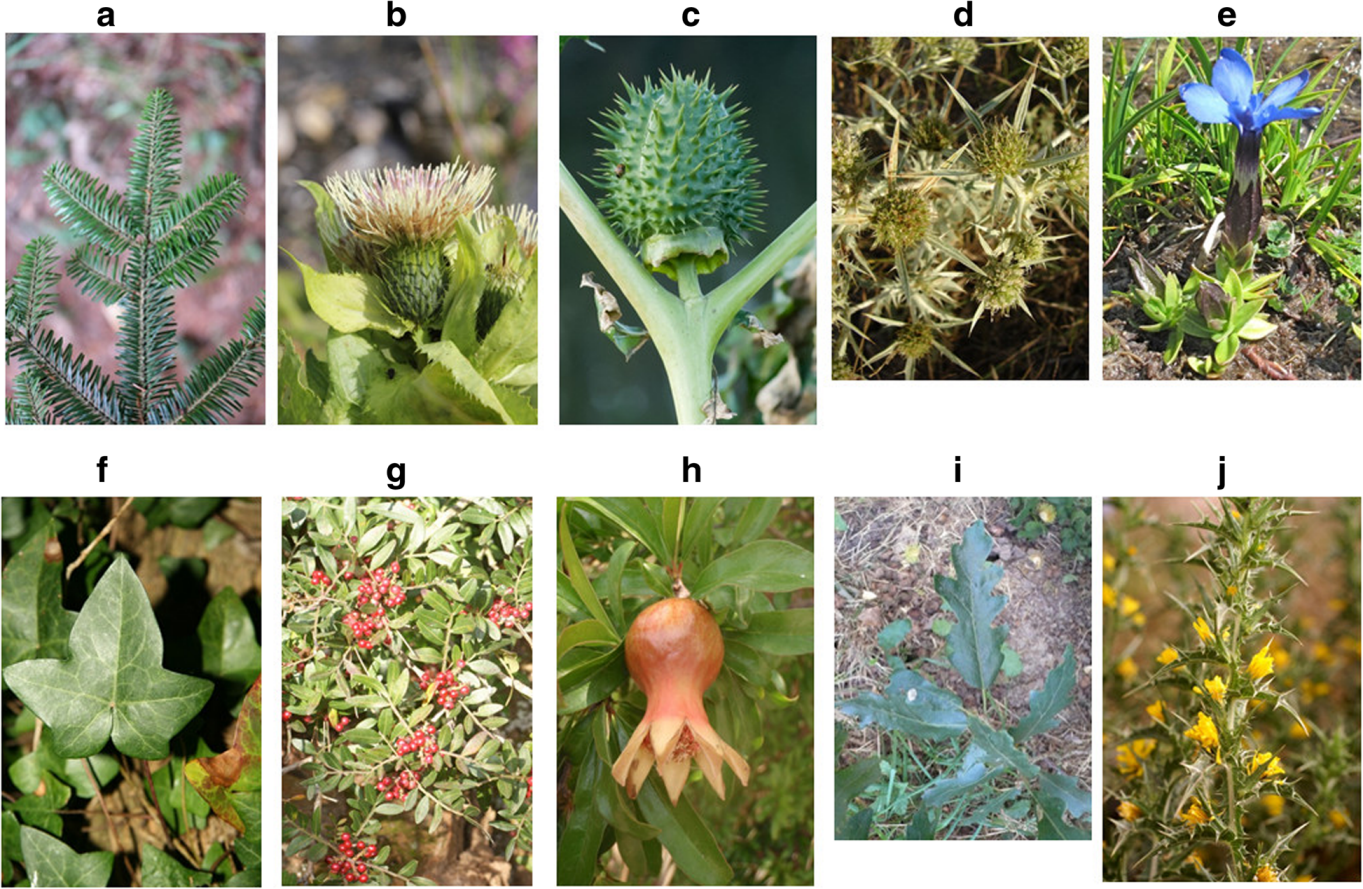
Images of different species used in the PlantCLEF (PC) dataset: **a** *Abies alba* Mill., **b** *Cirsium oleraceum* (L.) Scop., **c** *Datura stramonium* L., **d** *Eryngium campestre* L., **e** *Gentiana verna* L., **f** *Hedera helix* L., **g** *Pistacia lentiscus* L., **h** *Punica granatum* L., **i** *Quercus cerris* L., **j** *Scolymus hispanicus* L
- Herbarium1K (H1K): this dataset covers 1204 species, 918 of which are included in the 1000 species of the PlantCLEF dataset. Obtained through iDigBio, the dataset contains 202,445 images for training and 51,288 for testing. All images have been resized to a width of 1024 pixels and their height proportionally, given the huge resolutions used in herbarium images. Figure 6 shows a random sample taken from this dataset. This is an unbalanced dataset.

**Fig. 6:**
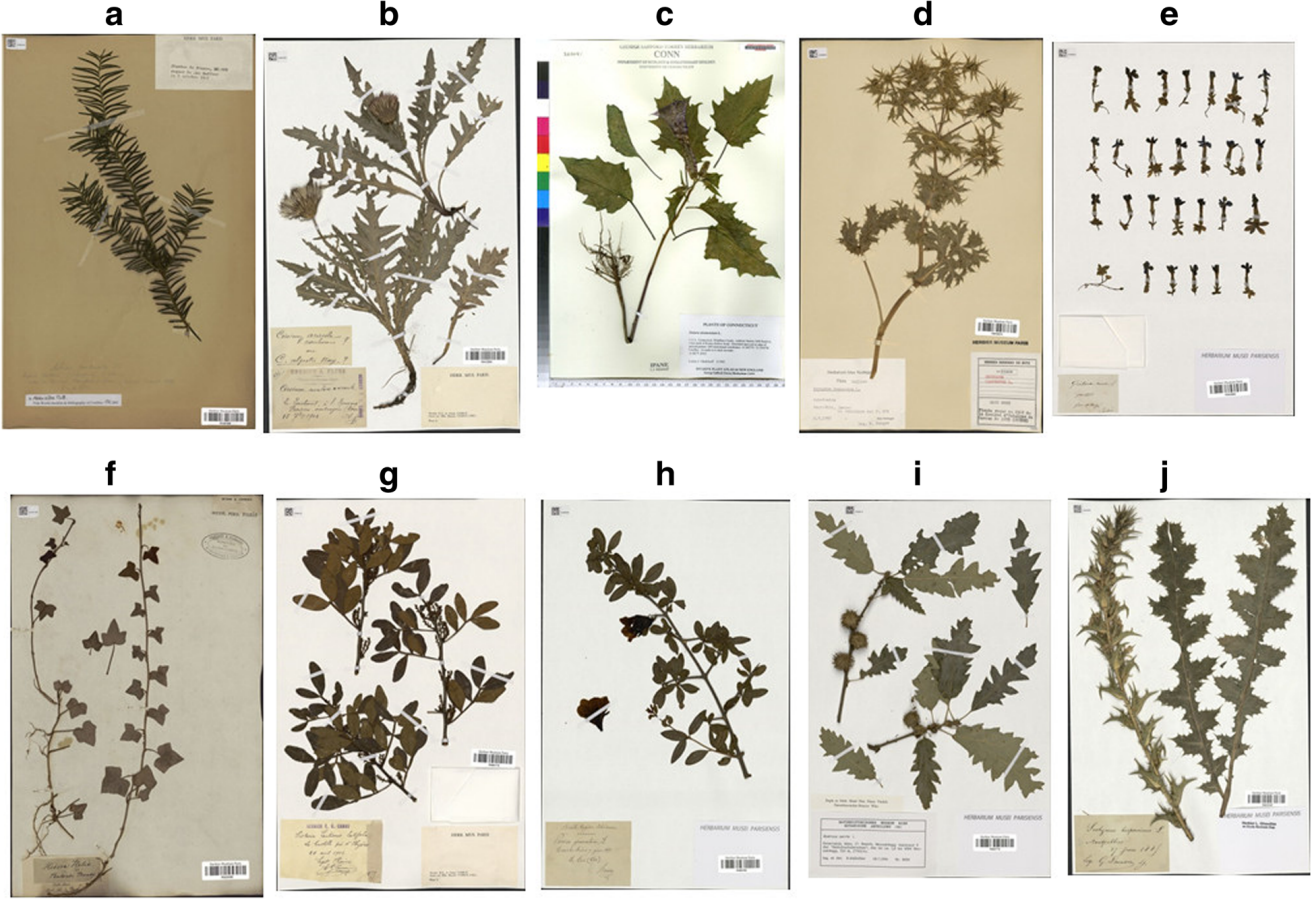
Ten herbarium sheet images used in the Herbaria1K (H1K) dataset: **a** *Abies alba* Mill, **b** *Cirsium oleraceum* (L.) Scop, **c** *Datura stramonium* L, **d** *Eryngium campestre* L, **e** *Gentiana verna* L, **f** *Hedera helix* L, **g** *Pistacia lentiscus* L, **h** *Punica granatum* L, **i** *Quercus cerris* L, **j** *Scolymus hispanicus* L
- ImageNet (I): ImageNet is arguably the image dataset most used by the machine learning research community. It contains 1000 generalist classes and more than a million images [40]. It is the de facto standard for pre-training deep learning models. We use only the weights of a trained model with this dataset for transfer learning proposes.

### Avoiding bias

To avoid biases in the experiments, we separated the datasets in a special way for training and testing. For herbarium datasets H255 and H1K, data was separated so that sheets of the same species that were collected by the same collector were not permitted to enter both the training and testing sets. For the CR dataset, we separated the data so that images of different leaves from each specimen are present in either the training or the testing set, but not in both. For the PlantCLEF (PC) dataset, we did this too at the observation level. So, no same observation is present in both training and testing subsets. These measures lead to more realistic and unbiased training/testing scenarios although they also lead to lower accuracy rates.

### Image pre-processing

All datasets were normalized to an uniform size of 256 by 256 pixels without any other type of pre-processing. This is the current state-of-the-art resolution as deep learning models are intensive in computing.

## Experiments and results

All experiments measured the top-1 and top-5 accuracy of the trained deep learning model under different circumstances, i.e., herbarium specimens classification (“Herbarium specimen classification” section, Table 3), transfer learning across herbarium data from different regions (“Cross-Herbaria transfer learning” section, Table 4), and transfer learning from herbarium data to non-dried plant images (“Transfer learning from herbarium to non-dried plant images” section, Table 5).

**Table 3:** Results of the experiments related to herbarium specimens classification

Experiment	Initialization	Training	Testing	Top-1 accuracy	Top-5 accuracy
Costa-Rica Flora					
*R*.*H*255.*H*255	Random	Herbarium255	Herbarium255	0.585	0.771
*I*.*H*255.*H*255	ImageNet	Herbarium255	Herbarium255	0.703	0.852
France Flora					
*R*.*H*1*K*.*H*1*K*	Random	Herbarium1K	Herbarium1K	0.726	0.871
*I*.*H*1*K*.*H*1*K*	ImageNet	Herbarium1K	Herbarium1K	0.796	0.903

**Table 4:** Results of the experiments related to cross-herbarium transfer learning

Experiment	Initialization	Training	Testing	Top-1 accuracy	Top-5 accuracy
Cross-herbaria Transfer learning (France to Costa-Rica)					
*H*1*K*.*H*255.*H*255	Herbarium1K	Herbarium255	Herbarium255	0.693	0.842
*H*1*K*_*I*_.*H*255.*H*255	ImageNet+Herbarium1K	Herbarium255	Herbarium255	0.745	0.872

**Table 5:** Results of the experiments related to transfer learning from Herbarium to non-dried plant images

Experiment	Initialization	Training	Testing	Top-1 accuracy	Top-5 accuracy
CRLeaves Baselines					
*R*.*C* *R*.*C* *R*	Random	CRLeaves	CRLeaves	0.37	0.50
*I*.*C* *R*.*C* *R*	ImageNet	CRLeaves	CRLeaves	0.51	0.61
CRLeaves using transfer learning from herbarium data					
*H*255.*C* *R*.*C* *R*	Herbarium255	CRLeaves	CRLeaves	0.416	0.542
*H*255_*I*_.*C* *R*.*C* *R*	ImageNet,Herbarium255	CRLeaves	CRLeaves	0.491	0.590
PlantCLEF Baselines					
*R*.*P* *C*.*P* *C*	Random	PlantCLEF	PlantCLEF	0.334	0.566
*I*.*P* *C*.*P* *C*	ImageNet	PlantCLEF	PlantCLEF	0.523	0.726
PlantCLEF using transfer learning from herbarium data					
*H*1*K*.*P* *C*.*P* *C*	Herbarium1K	PlantCLEF	PlantCLEF	0.273	0.498
*H*1*K*_*I*_.*P* *C*.*P* *C*	ImageNet,Herbarium1K	PlantCLEF	PlantCLEF	0.425	0.661

For each of these experiments, table columns are defined as follows:

- *Experiment*: the name of the experiment. It follows the 〈*Initialization*〉.〈*Training*〉.〈*Testing*〉 pattern, using the dataset acronyms already discussed. For example, *I*.*P* *C*.*P* *C* means the initialization of weights was done by pre-training the network on ImageNet, then fine-tuning it on PlantCLEF training set, and finally testing it with PlantCLEF test set. Similarly, *R*.*P* *C*.*P* *C* has almost the same meaning, but the initialization was Random (i.e., no tranfer learning was used). Also, we use index *I* to mean that at the very beginning the weights of ImageNet were used. For example, *H*1*K*_*I*_.*P* *C*.*P* *C* means the transfer learning was progressive, done from ImageNet, to Herbarium1K, to PlantCLEF, and tested with PlantCLEF data.
- *Initialization*: weights used to initialize the model.
- *Training*: training set used (e.g., Herbarium255 training set, PlantCLEF training set, etc.)
- *Testing*: test set used (e.g., Herbarium255 test set, PlantCLEF test set, etc.)
- *Top-1/Top-5*: accuracy achieved with top-1 and top-5 best predictions, respectively.

### Herbarium specimen classification

These experiments aim at assessing the feasibility of using a deep learning system dedicated to herbarium specimen identification at a realistic scale (255 species from Costa-Rica in Herbarium255 and 1K species from France in Herbarium1K). Herbarium255 was divided in 70% training data and the rest 30% as test data used for computing the top-1 and top-5 classification accuracy. Herbarium1K was divided in 80 and 20% respectively, to keep the proportion of the data provided by the PC challenge. The separation was done by species, and within each species, no collector was shared by the training and testing sets to avoid bias in the data. The following four experiments were conducted:

- *R*.*H*255.*H*255: The neural network was initialized randomly, trained on the Herbarium255 training set (70%), and tested on the Herbarium255 test set (30%).
- *I*.*H*255.*H*255: The neural network was pre-trained on the generalist dataset ImageNet to initialize the weights, fine-tuned on the Herbarium255 training set (70%), and tested on the Herbarium255 test set (30%).
- *R*.*H*1*K*.*H*1*K*: The neural network was initialized randomly, trained on the Herbarium1K training set (80%), and tested on the Herbarium1K test set (20%).
- *I*.*H*1*K*.*H*1*K*: The neural network was pre-trained on the generalist dataset ImageNet to initialize the weights, fine-tuned on the Herbarium1K training set (80%), and tested on the Herbarium1K test set (20%).

Table 3 synthesizes the results of these experiments. A first clear result is that the best accuracies are achieved when ImageNet was used for the initialization step rather than using random weights. This means that herbarium data alone is not sufficient to train the neural network from scratch and that transfer learning from another dataset is significant.

Secondly, when using transfer learning, the achieved accuracies are impressive compared to previous work. We actually obtain similar top-1 accuracies than the recent study of Unger et al. [11] (73% and 84% on 26 and 17 species, respectively) whereas our classifier is tested (and trained) on one to two orders of magnitude more species. In particular, with a 90% top-5 accuracy for the Herbarium1K dataset, these experiments show that a real-world system to help with herbarium sheet classification is clearly doable.

Thirdly, the slightly better performance on the Herbarium1K dataset compared to to the Herbarium255 dataset is probably related to the fact that the average number of images per species in the training set is much higher (207.13 images per species in Herbarium1K vs. 43.42 images per species in Herbarium255). This would also explain why the gain due to transfer learning is higher for Herbarium255. As the targeted classes (i.e. species) are illustrated by less images, the low-level layers of the network benefit more from training on more visual contents beforehand.

### Cross-Herbaria transfer learning

Experiments *H*1*K*.*H*255.*H*255 and *H*1*K*_*I*_.*H*255.*H*255, as shown in Table 4, compare how prediction works on Herbarium255 (Costa Rica) after transfer learning from Herbarium1K (France). This is important because it provides insights on the possibility of training a deep learning model on a region of the world and use that knowledge in predictions for a different region, particularly for regions where there are not that many herbarium specimen images. In summary, we conducted the following two experiments:

- *H*1*K*.*H*255.*H*255: The neural network was pre-trained on the Herbarium1K dataset to initialize the weights, fine-tuned on the Herbarium255 training set (70%), and tested on the Herbarium255 test set (30%).
- *H*1*K*_*I*_.*H*255.*H*255: The neural network was pre-trained on ImageNet and then on Herbarium1K before being fine-tuned on the Herbarium255 training set (70%), and finally tested on the Herbarium255 test set (30%).

As shown in Table 4 the results are very promising. By comparing experiment *H*1*K*_*I*_.*H*255.*H*255 with experiment *I*.*H*255.*H*255 (replicated from Table 3), Herbarium255 prediction improves by 4.1% on top-1 accuracy and by 1.9% for top-5 if Herbarium1K is used for transfer learning. It is likely that using the whole iDigBio repository for transfer learning instead of Herbarium1K could give even better results but this is beyond the scope of this paper.

If we compare experiment *H*1*K*.*H*255.*H*255 with *I*.*H*255.*H*255, the accuracy is almost the same, suggesting that transfer learning from ImageNet only performs similarly to transfer learning from Herbarium1K only. This is good news in the sense that Herbarium1K has much less images than ImageNet, which proves that a dataset smaller than ImageNet but specialized in a specific domain can be as effective in terms of transfer learning.

Finally, by comparing experiment *H*1*K*.*H*255.*H*255 with *R*.*H*255.*H*255 (replicated from Table 3), we also get an improvement in the accuracy of 10.7% for top-1 and 7% for top-5, suggesting it is way better to use a herbarium dataset from another region for transfer learning instead of just doing random weights initially.

### Transfer learning from herbarium to non-dried plant images

These experiments are meant to measure if using herbarium images for progressive transfer learning is useful on other data types, in particular field images and non-dried leaf scans. Therefore, we conducted the following experiments:

- *R*.*C* *R*.*C* *R*: The neural network was initialized randomly, trained on the Costa-Rica leaf scans training set (70%) and tested on the Costa-Rica leaf scans test set (30%).
- *I*.*C* *R*.*C* *R*: The neural network was pre-trained on the generalist dataset ImageNet to initialize the weights, fine-tuned on the Costa-Rica leaf scans training set (70%) and tested on the Costa-Rica leaf scans test set (30%).
- *H*255.*C* *R*.*C* *R*: The neural network was pre-trained on the Herbarium255 dataset to initialize the weights, fine-tuned on the Costa-Rica leaf scans training set (70%) and tested on the Costa-Rica leaf scans test set (30%).
- *H*255_*I*_.*C* *R*.*C* *R*: The neural network was pre-trained on ImageNet and then on Herbarium255 before being fine-tuned on the Costa-Rica leaf scans training set (70%) and finally tested on the Costa-Rica leaf scans test set (30%).
- *R*.*P* *C*.*P* *C*: The neural network was initialized randomly, trained on the PlantCLEF training set (80%) and tested on the PlantCLEF test set (20%).
- *I*.*P* *C*.*P* *C*: The neural network was pre-trained on the generalist dataset ImageNet to initialize the weights, fine-tuned on the PlantCLEF training set (80%) and tested on the PlantCLEF test set (20%).
- *H*1*K*.*P* *C*.*P* *C*: The neural network was pre-trained on the Herbarium1K dataset to initialize the weights, fine-tuned on the PlantCLEF training set (80%) and tested on the PlantCLEF test set (20%).
- *H*1*K*_*I*_.*P* *C*.*P* *C*: The neural network was pre-trained on ImageNet and then on Herbarium1K before being fine-tuned on the PlantCLEF training set (80%) and finally tested on the PlantCLEF test set (20%).

Table 5 synthesises the results of these experiments. The main conclusion is that initializing the models with ImageNet always results in better accuracy for all experiments. If we compare experiments *R*.*C* *R*.*C* *R* and *H*255.*C* *R*.*C* *R*, fine tuning over herbaria against the randomly initialized baseline offers an accuracy increase of 4.7 and 3.8% for top-1 and top-5 respectively. By comparing experiments *R*.*C* *R*.*C* *R* and *H*255_*I*_.*C* *R*.*C* *R*, the increase goes up to 12.1 and 8.6% respectively, but still, it is less effective than fine-tuning directly from the ImageNet dataset (*I*.*C* *R*.*C* *R*). This result is aligned with previous evaluations in the literature (see e.g. [30, 31]). It confirms that models trained on a big generalist dataset such as ImageNet can be used as generic feature extractors for any domain-specific task. On the contrary, the visual features learned on Herbarium255 are more specific to herbarium content and do generalize less well to the leaf scans classification task (even if Herbarium255 and CRLeaves cover the same species). This is coherent with the conclusions of Tomaszewski et al. [27] that leaf shape changes during the drying process and that shape analyses should be performed on datasets containing only dried or fresh leaves.

**Fig. 7:**
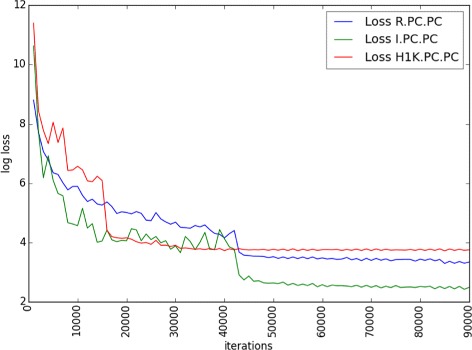
Comparison of losses of *R*.*P* *C*.*P* *C*, *I*.*P* *C*.*P* *C* and *H*1*K*.*P* *C*.*P* *C* experiments

The results obtained on the PlantCLEF dataset suggest that it is even less possible to transfer knowledge from herbarium to field images (in particular, wild flower images, which is the most represented type of view in the PlantCLEF dataset). By comparing the results of experiment *R*.*P* *C*.*P* *C* and *H*1*K*.*P* *C*.*P* *C*, we can actually notice that the accuracy decreases by 6.1% for the top-1 and 6.8% for the top-5. If we compare *I*.*P* *C*.*P* *C* with *H*1*K*_*I*_.*P* *C*.*P* *C*, the decrease reaches 9.8%. This means that the visual features learned from the herbarium data are even worse than random features for the initialization of the network. To better understand the reason for this phenomenon, we plotted in Fig. 7 the evolution of the loss function of the network during training (for experiments *R*.*P* *C*.*P* *C*, *I*.*P* *C*.*P* *C* and *H*1*K*.*P* *C*.*P* *C*). It shows that using the *H*1*K*-based initialization causes the network to converge quickly to a stable but worse solution than when using the random or the ImageNet-based initialization. Our interpretation is that the stochastic gradient descent is blocked into a saddle point close to a local minimum. This is probably due to the fact that the visual features learned on the herbarium data are somehow effective in classifying the field images, but far away from the optimal visual features that should be learned. The visual aspect of a herbarium image is indeed very different from a picture of a plant in natural conditions. Several phenomena affect the transformation of the plant sample during the drying process. There is first a strong variation of the colors of the plant, indeed most of the dry leaves have a brown instead of a green color when they are fresh, flower and fruit colors are also strongly impacted. Furthermore, herbarium specimens have often an overlap of their leaves with flowers and fruits that makes difficult the automated identification of the object of interest in the herbarium image. 3D objects such as fruits and flowers are also completely transformed when they are pressed. These transformations are most probably the reasons why transfer learning from herbarium images to field data isn’t effective.

## Discussion and conclusions

This study is, to our knowledge, the first one that analyzes a big dataset with thousands of specimen images from herbaria and uses deep learning. Results show the potential of deep learning on herbarium species identification, particularly by training and testing across different herbarium datasets. This could potentially lead to the creation of a semi, or even fully, automatic system to help taxonomists and experts do their annotation, classification, and revision work at herbarium.

In particular, we showed that is possible to use a herbarium image dataset from one region of the world to do transfer learning to another region, even when the species do not match. This indicates that a deep learning approach could be used in regions that do not have lots of herbarium images. On the negative side, we did show that it is not beneficial to do transfer learning from herbarium data to leaf scan pictures and it is even counterproductive to do transfer learning from herbarium data to field images. This confirms some previous studies in the literature that concluded that the observable morphological attributes can change significantly with the drying process. Additionally, the particular unnatural layout of plants and their parts on herbarium sheets may also have a negative effect.

It is worth trying to apply some pre-processing on the herbarium datasets for further experimentation, particularly to get rid of handwritten tags and other visual noise present in the herbarium sheets. Additionally, as per results on only herbarium data, it would be a good idea to start working on a model whose hyperparameters, architecture and data augmentation are thought for herbarium in particular, to maximize accuracy for a system dedicated to herbarium in mind. More experiments with bigger leaf datasets are recommended, since some viability of using herbarium for fine tuning on leaf images was observed. Concerning the question of how herbarium data could be useful for field images classification, we believe we should rather try to model the drying process itself typically by learning a transfer function between a representation space dedicated to herbarium images and another one dedicated to field images. In order to improve the accuracy in future experiments, an option is to explore the taxonomy as a class hierarchy. Several others possibilities could potentially improve transfer learning between herbarium images and images of plants in the field. Herbarium annotation (with tags on what is possible to see in the image of the specimen) could be a first important step of progress for the computer vision community. Indeed, if we are able for the same species to use images of herbarium and plant in the field that contain the same visual information (both in flower, or with leaves for example), we will be able to better understand contexts in which transfer learning failed or potentially be improved. Herbarium visual quality evaluation could be also of a great interest. Indeed, some herbarium specimens can be really precious for the botanical community, but if the plant sample in the image is too old and damaged, this specimen will be of poor interest for automated species identification. The individual image quality evaluation could be very useful to weight the use of each images during the learning phase on training datasets.

Finally, based on our results, we believe that the development of deep learning technology based on herbarium data, together with the recent recognition of e-publication in the International Code of Nomenclature [41] will also contribute to significantly increase the volume of descriptions of new species in the following years.
